# UHPLC–MS/MS Method for the Simultaneous Quantification of 12 Antiretroviral Drugs in Human Plasma Using Dried Sample Spot Devices: Development, Validation, and Stability Evaluation

**DOI:** 10.3390/pharmaceutics18040513

**Published:** 2026-04-21

**Authors:** Sara Soloperto, Elisa Martina, Alice Palermiti, Elisa Barnini, Greta Sabbia, Gianluca Bianco, Martina Billi, Camilla Martino, Alessandra Manca, Marco Simiele, Jessica Cusato, Antonio D’Avolio, Amedeo De Nicolò

**Affiliations:** 1 Laboratory of Clinical Pharmacology and Pharmacogenetics, Department of Medical Sciences, University of Turin, 10149 Turin, Italy; elisa.martina@unito.it (E.M.); alice.palermiti@unito.it (A.P.); elisa.barnini@unito.it (E.B.); greta.sabbia@edu.unito.it (G.S.); gianluca.bianco@aslcittaditorino.it (G.B.); martina.billi@unito.it (M.B.); camilla.martino@unito.it (C.M.); alessandra.manca@unito.it (A.M.); jessica.cusato@unito.it (J.C.); antonio.davolio@unito.it (A.D.); amedeo.denicolo@unito.it (A.D.N.); 2 CoQua Lab s.r.l., 10149 Turin, Italy; marco.simiele@coqualab.it

**Keywords:** antiretrovirals, therapeutic drug monitoring, dried plasma spot, long acting, mass spectrometry, liquid chromatography, microsampling, pharmacokinetics

## Abstract

**Background/Objectives**: In several contexts, Dried Sample Spot Devices (DSSDs) offer a convenient and safe alternative for sampling, storage, and shipment, allowing the transport and storage of biological samples at room temperature, reducing shipment costs and improving access to diagnostics in faraway sites. This can be pivotal for the use of the therapeutic drug monitoring of anti-HIV treatment: therefore, this study aimed to develop and validate a UHPLC–MS/MS method for the simultaneous quantification of 12 antiretroviral drugs, including the recently introduced long-acting agents, in Dry Plasma Spots (DPSs). **Methods**: First, 100 µL of plasma sample and 100 µL of internal standard solution were spotted on each DSSD. After complete drying, DPSs were added with an acidifying solution (ammonium acetate buffer pH 4), and then, each sample underwent extraction with hexane-dichloromethane 50:50 (*v*/*v*). After tumbling, the organic phase was evaporated and reconstituted for injection. An Acquity UPLC HSS T3 1.8 µm, 2.1 × 150 mm column at 50 °C enabled separation, performed using H_2_O + F.A. 0.05% (phase A) and ACN + F.A. 0.05% (phase B) as the mobile phase in gradient elution mode, for a total run time of 15 min. **Results:** The method was validated over the clinically relevant concentration ranges. For all quality control levels, accuracies ranged from 98.2% to 114.1%, and intra-day and inter-day RSD values ranged from 2.7% to 9.7% and 5.2% to 13.9%, respectively. All analytes demonstrated satisfactory short- and long-term stability in DPSs, confirming the suitability of shipment and storage at room temperature. **Conclusions**: The method demonstrated robustness and reproducibility in accordance with FDA and EMA guidelines. It ensures satisfactory accuracy and rapid analysis, supporting its application in clinical practice, including for monitoring the newest long-acting drugs.

## 1. Introduction

Although significant developments in antiretroviral therapy (ART) have successfully transformed HIV from a swift and lethal infection into a persistent ailment through the implementation of potent combined antiretroviral therapy (cART) [1], the United Nation Programme on HIV and AIDS (UNAIDS) reported that, in 2023, 39.9 million people were living with HIV, and 1.3 million people were newly infected with HIV [2]. These numbers reveal the constant necessity to develop new drugs and improve those already existing.

In this context, Therapeutic Drug Monitoring (TDM) represents a useful tool for anti-infectives monitoring, with its application becoming frequently used in clinical settings.

Traditionally, TDM assays for antiretroviral drugs (ARVs) have been performed using liquid plasma or serum samples from venous blood sampling. However, this seemingly simple approach has some limitations when applied to certain settings [3]. Nowadays, few laboratories are able to perform TDM analysis (especially regarding recently introduced drugs, e.g., cabotegravir), forcing many hospitals in many countries to incur substantial costs for the storage and dry-ice shipping of samples to specialized laboratories abroad; these currently represent two relevant limitations to widespread TDM practice [4].

In this scenario, microsampling combined with high-sensitivity analytical techniques, like liquid chromatography coupled with tandem mass spectrometry (LC-MS/MS), represents an efficient option for quantifying analytes in small-volume samples with good sensitivity, offering an application opportunity in clinical pharmacology and TDM [5]. This approach provides several advantages: minimal invasiveness, the use of small volumes, a lower risk of contamination, a lower biological risk, better stability, and easy storage [3].

In this context, Dried Sample Spot Devices (DSSDs), whether Dried Blood Spots (DBSs) or Dried Plasma Spots (DPSs), have already been employed for other classes of drugs (e.g., antipsychotics, antiepileptics, and antifungals) [6,7,8]. In addition, different protocols were developed to measure HIV-1 viral loads by using DBSs [9,10,11,12].

DSSDs comprise special devices of different materials (e.g., paper and glass fibre) on which a few microliters of whole blood or plasma are spotted and dried: these can be subsequently stored and shipped, away from light, at room temperature, according to the molecule’s stability.

In 2018, Mark D. Lim, from the Global Health Division, reported that DSSDs like DBSs offer several logistical advantages for remote health and surveillance programs, particularly for screening and surveying hard-to-reach populations [13], allowing a major lowering of the shipping costs in particular as they are allowed to be shipped at room temperature.

Nowadays, in many therapeutic contexts, long-acting regimens are increasingly important. In particular, in the context of cART, long-acting injectable formulations (LAI) received European Medicine Agency (EMA) approval in 2020 [14,15]. The intramuscular administration of rilpivirine (RPV) and cabotegravir (CAB) is becoming more common, as, in many trials, switching to long-acting therapy is demonstrated as not being inferior to the continuation of current oral cARTs among adults with virologically suppressed HIV-1 infection [16,17,18]. Moreover, the use of LAI has been proven to improve therapeutic adherence as well as to enhance treatment satisfaction in people living with HIV (PLWH) [19].

Previous studies described the quantification methods for ARVs in DSSDs, but all of them focused on DBS [20,21,22] and did not include recently approved drugs, thus leading to results that are outdated and no longer useful in the current scenario.

Only recently, one paper described an LC-MS/MS method for the analysis of long-acting injectable molecules (RPV and CAB) in DBS, although this outlined the limitations regarding uncertainty on the estimated plasma concentrations, particularly due to the effect of inter-patient variability in the hematocrit and in the blood-to-plasma ratio [23].

In this regard, using DPSs theoretically overcomes these issues, improving the reliability of drug quantification, while keeping the advantages in terms of cheaper shipping, storage and improved biological safety. For these reasons, our team previously reported a validated HPLC-MS method to quantify a panel of 9 ARVs in DPSs which, although are mostly outdated and are falling out of use [4].

Therefore, the aim of this work is to illustrate an updated UHPLC-MS/MS method for the quantification of 12 different antiretroviral drugs administered for HIV treatment using DPS, including the majority of the ARVs currently included in the WHO guidelines, excluding drugs of a nucleosidic nature (NRTIs). The proposed updated ARVs panel is as follows: integrase strand transfer inhibitors (INSTIs) including raltegravir (RAL), bictegravir (BIC), cabotegravir (CAB), and dolutegravir (DTG); non-nucleoside reverse-transcriptase inhibitors (NNRTIs), including rilpivirine (RPV), doravirine (DOR), temsavir (TEM), efavirenz (EFV), and nevirapine (NVP); and protease inhibitors (PIs), including darunavir (DRV), ritonavir (RTV), and atazanavir (ATV).

The validation process includes a thorough stability evaluation at room temperature up to 1 month for all the analytes spotted on filters, in order to assess the eligibility for shipping by ordinary mail, and the stability for 1 week at room temperature followed by 1 week at 4 °C to simulate shipping and in-laboratory storage conditions before analysis.

## 2. Materials and Methods

### 2.1. Chemicals

Acetonitrile (ACN) and methanol (MeOH) of HPLC quality were acquired from VWR (Milan, Italy). A Milli-DI system in combination with a Synergy 185 system from Millipore (Milan, Italy) was employed to generate HPLC-grade water (H_2_O). Ammonium Acetate, Formic Acid (F.A.), Dimethyl sulfoxide (DMSO), 2-Propanol, Dichloromethane, Ortho-Phosphoric Acid 85% for HPLC, Hexane, and Acetic Acid were purchased from Sigma-Aldrich Corporation (Milan, Italy).

Powders of NVP (purity 99.93%), RPV (purity 99.71%), EFV (purity 99.84%), DOR (purity 99.3%), TEM (purity 98.06%), DRV (purity 99.84%), RTV (purity 99.92%), ATV (purity 99.87%), RAL (purity 98.13%), BIC (purity 99.24%), CAB (purity 99.72%), and DTG (purity 99.98%) were provided by Medchem Express (Bergkällavägen, Sollentuna, Sweden).

All isotope-labelled molecules used as internal standards (IS) ([^13^C,^2^H_3_]-RTV (purity 95.2%; isotopic enrichment 99.0% ^13^C and 99.8% ^2^H_3_), [^13^C_6_]-EFV (purity 99.1%; isotopic enrichment 99.0% ^13^C), [^2^H_6_]-ATV (purity 96.9%; isotopic enrichment 98.9% ^2^H_6_), [^13^C,^2^H_5_]-DTG (purity 100.0%; isotopic enrichment 99.5% ^13^C and 98.0% ^2^H_5_), [^13^C, ^2^H_2_, ^15^N]-CAB (purity 97.1%; isotopic enrichment 99.9% ^13^C, 99.6% ^2^H_2_, and 99.9% ^15^N), [^13^C_6_]-DOR (purity 95.2%; isotopic enrichment 99.0% ^13^C_6_), [^13^C, ^2^H_2_, ^15^N]-BIC (purity 99.8%; isotopic enrichment 99.0% ^13^C, 99.3% ^2^H_2_, and 98.0% ^15^N), [^13^C_6_]-DRV (purity 99.1%; 99.4% ^13^C_6_), and [^13^C_6_]-RPV (purity 95.0%; isotopic enrichment 99.0% ^13^C_6_)) were provided by AlsaChim (Illkirch-Graffenstaden, France). As indicated by the Certificate of Analysis (CoA) for each drug, all powders were kept at −20 °C (with exception for BIC and RTV which were stored at +4 °C) in the dark to avoid any potential deterioration.

### 2.2. Stock Solutions, Internal Standards, Standards, and Quality Controls

Stock solutions for analytes (2 mg/mL) and for isotope-labelled molecules (1 mg/mL) were prepared as follow: NVP, RAL, RTV, EFV, DTG, ATV, [^13^C,^2^H_3_]-RTV, [^13^C_6_]-EFV, [^13^C,^2^H_5_]-DTG, and [^13^C_6_]-DRV stock solutions in a mixture of H_2_O:MeOH 10:90 (*v*/*v*); CAB, DOR, TEM, [^13^C_6_]-RPV, [^13^C, ^2^H_2_, ^15^N]-CAB, and [^13^C_6_]-DOR stock solutions were prepared in 100% DMSO; DRV and RPV stock solutions were prepared in DMSO: MeOH 50:50 (*v*/*v*); BIC stock solution was prepared in H_2_O:2-propanol 25:75 (*v*/*v*); and the isotope-labelled molecule stock solutions of [^13^C, ^2^H_2_, ^15^N]-BIC and [^2^H_6_]-ATV were prepared in 100% ACN. Before being used, all stock solutions were stored at −80 °C according to the expiry date reported on CoAs. “Plasma for industry use”, dedicated to the production of hemodiagnostics and plasma-derived products, was kindly supplied by the blood bank of the “Città della Salute e della Scienza” (Turin, Italy) and used as “blank” (analyte-free) plasma for the preparation of calibrators and quality controls.

After independently spiking “blank” plasma with stock solutions, single aliquots of calibrator and Quality Controls (QCs) were created and stored at −20 °C.

Nominal concentrations for calibration ranges and QC levels for each analyte will be provided in detail within Section 3.2.

In order to prepare the IS working solution, at each analytical session, the isotope-labelled molecules were diluted in a 70:30 (*v*/*v*) solution of H_2_O and MeOH acidified with 0.01% ortho-phosphoric acid at an appropriate concentration within the calibration range for each analyte. The IS working solution was then stored at +4 °C for one month.

### 2.3. Sample Preparation

Glass-fibre DSSDs were kindly supplied by CoQua lab s.r.l (Turin, Italy).

**Figure 1:**
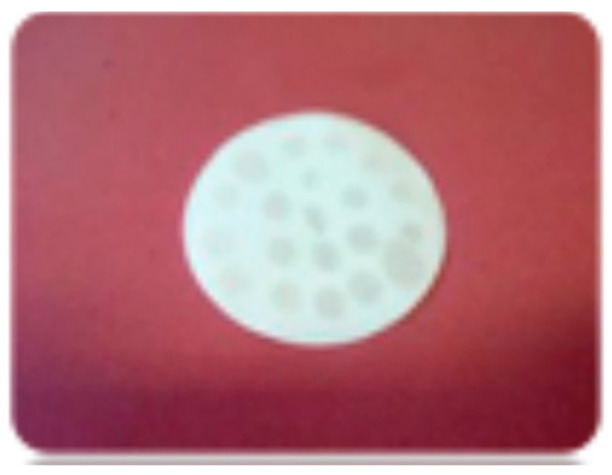
Dried Plasma Spot on DSSD (DPS-DSSD).

For the extraction process, 100 μL of plasma samples were spotted on DSSDs (Figure 1) and allowed to dry for 15 min at room temperature. Then, 100 μL of Internal Standard solution was then added and allowed to dry for further 15 min. The resulting DPSs were then folded and placed in 5 mL Eppendorf tubes, where 200 μL of ammonium buffer solution (ammonium acetate 2 mM added with acetic acid, pH 4) and 1800 μL of extraction solution (Hexane: Dichloromethane 50:50 (*v*/*v*)) were sequentially added.

After vortex mixing, samples were tumbled at 50 rpm for 15 min, and, after transferring to bacteriological tubes, they were evaporated at 50 °C into a vacuum concentrator (Labconco Corporation, Kansas City, MO, USA). Finally, the dry extracts were reconstituted with 1 mL of H_2_O:ACN 70:30 (*v*/*v*), transferred into glass vials, and 5 μL were, then, injected into the chromatographic system.

### 2.4. UPLC–MS/MS Instruments and Chromatographic Conditions

The UHPLC-MS/MS system was composed of an LX-50 ^®^ UHPLC coupled with a QSight 220^®^ tandem mass spectrometer (Perkin Elmer, Shelton, CT, USA).

The chromatographic separation was performed with an Acquity^®^ UPLC HSS T3 1.8 µm, 2.1 × 150 mm column (Waters, Milford, MA, USA), kept at 50 °C with a column oven: H_2_O + F.A. 0.05% (phase A) and ACN + F.A. 0.05% (phase B) were employed in gradient elution mode. The total chromatographic run time was 15 min, with a constant flow rate of 0.4 mL/min. The initial mobile phase composition consisted of 30% phase B: a detailed description of the chromatographic gradient over time is reported in Table S1 (Supplementary Materials).

The instrument was set in positive electrospray ionization configuration (ESI+) for all analytes except for EFV, and its stable isotope linked internal standard (SIL-IS), which required a negative electrospray ionization configuration (ESI−). General source settings are reported in Table S2 (Supplementary Materials).

Analyte-specific mass parameters with the Multiple Reaction Monitoring (MRM) ion traces for each molecule are reported in Table 1.

### 2.5. Accuracy, Precision, and Limit of Quantification

During 6 validation sessions, 5 intra-day replicates at 4 different quality controls (QC H, QC M, QC L, and LLOQ) concentrations were analyzed to assess accuracy and precision of the method (15 samples per validation session).

To ensure accurate evaluation of the reproducibility of method performance across different plasma lots, which could be hindered by variable recovery or matrix effect, different plasma lots were used to prepare the calibration curves at each validation session.

Intra-day (within session) and inter-day (between sessions) precision were reported as relative standard deviations (RSDs). Accuracy was calculated as the ratio between the mean determined value and the nominal concentration. Intra- and inter-day accuracy values were evaluated for each analyte.

The lowest limits of quantification (LLOQs) corresponded to the lowest concentration values of each calibration curve.

According to FDA and EMA recommendations, percentages of bias from the nominal concentration (a measure of inaccuracy) and RSDs at the LLOQ for each analyte must be less than 20% [24,25,26].

### 2.6. Matrix Effect

The matrix effect was evaluated by comparing peak areas of five intra-day replicates of blank plasma extracts, spiked after the extraction (“post-extraction addition” method) at concentrations corresponding to QC-H, QC-M, and QC-L in six plasma lots, with peak areas obtained from direct injection of pure solvents (H_2_O:ACN 70:30 (*v*/*v*)) spiked at the same analytes’ concentrations [24,25].

Furthermore, the IS-normalized (IS-n) matrix effect was assessed, as described in previous works [27], to evaluate the capability of the IS to compensate for matrix effect. The acceptance limit for the IS-normalized matrix effect was set at 15% of RSD.

### 2.7. Recovery

Similar to the matrix effect, recovery was evaluated by comparing five intra-day replicates peak areas obtained from the analysis of QC-H, QC-M, and QC-L plasma samples (spiked before extraction) from six different plasma lots with those from “post-extraction spiked” samples (see Section 2.6).

For the post-spiked samples, the “blank” plasma extracts were spiked at same theoretical drug concentrations of QC-H, QC-M, and QC-L, assuming 100% recovery. Analogously to the IS-n matrix effect, the IS-n recovery was evaluated to describe the capability of the IS to compensate for the variability in the recovery.

### 2.8. Stability

To assess analytes’ stability in DPS, plasma QCs were spotted on DSSDs and stored in foil bags with a desiccant for each device, away from light at room temperature until extraction to simulate the shipping conditions.

Due to the already well-known stability in liquid plasma of these analytes [28,29], stability evaluation in DPSs was performed at room temperature (24–25 °C) up to 1 month, to cover any shipping condition and possible delays, and up to 2 weeks, one at room temperature and one at 4 °C, to simulate a likely process of room-temperature shipping and refrigerated storage in receiving laboratories. Stability was reported as percentages of degradation at the three QC levels.

### 2.9. Method Comparison with Liquid Plasma Extraction and Incurred Samples’ Re-Analysis

After routine therapeutic drug monitoring on liquid plasma, anonymized leftover samples intended for destruction were used to test method performance in real-world conditions, according to the approval by the AOU “Città della Salute e della Scienza di Torino” Ethics Committee (protocol no. 0000248/2024) and in compliance with the Declaration of Helsinki. When possible (enough samples were received for a specific drug quantification), the results obtained from analysis in liquid plasma and DPS were compared through Passing–Bablok regression and Bland–Altman plot.

Moreover, according to the guidelines, these samples were re-evaluated in two independent analytical sessions on DPS to determine the “incurred samples precision” [26].

## 3. Results

### 3.1. Specificity and Selectivity

After chromatographic separation, all the analytes were retained according to their mass, pKa, and polarity properties [30,31,32,33,34,35,36,37,38,39,40,41].

**Figure 2:**
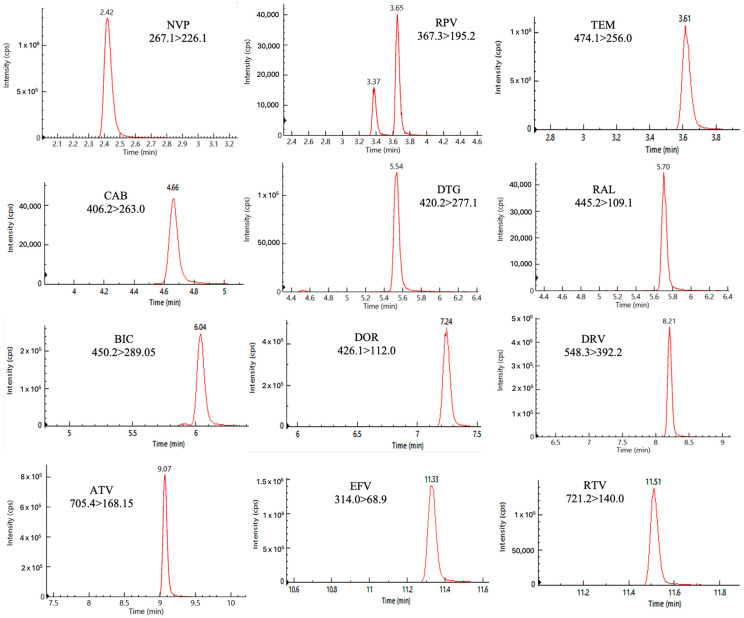
Chromatographic peaks of the quantifier ion traces of each analyte at the highest standard concentration.

Figure 2 shows chromatograms for each analyte, reported as absolute signal intensities, corresponding to the analysis of the highest calibration standard. The mean (standard deviation) retention times (RT, Figure 2) for the considered analytes are reported as follows: NVP 2.42 min (±0.05); TEM 3.61 min (±0.05); RPV 3.65 min (±0.05); CAB 4.66 min (±0.05); DTG 5.54 min (±0.05); RAL 5.70 min (±0.05); BIC 6.04 min (±0.05); DOR 7.24 min (±0.05); DRV 8.21 min (±0.05); ATV 9.07 min (±0.05); EFV 11.33 min (±0.05); and RTV 11.51 min (±0.05). No interfering peaks were detected in blank plasma samples at the RTs of the analytes.

To achieve data standardization, each analyte measured was paired with a corresponding SIL molecule, used as IS: [^13^C_6_]-RPV was used as IS for NVP, RPV, and TEM; [^13^C,^2^H_5_]-DTG for DTG; [^13^C, ^2^H_2_, ^15^N]-BIC for RAL and BIC; [^13^C_6_]-DRV for DRV; [^2^H_6_]-ATV for ATV; [^13^C_6_]-EFV for EFV; [^13^C,^2^H_3_]-RTV for RTV; [^13^C_6_]-DOR for DOR; and [^13^C, ^2^H_2_, ^15^N]-CAB for CAB.

In Figure 2, two peaks are appreciable for RPV, representing two different isomers: the first and less abundant peak is the (*Z*) isomer, which is generated following exposure to light and is considered as a known impurity; the second and more abundant peak is the (*E*) isomer, which is the active isomer and the real target for TDM [42,43].

No carry-over, crosstalk, nor interference were observed at the analytes’ RTs.

### 3.2. Accuracy, Precision, Linearity, and Limits of Quantification

The calibration parameters, including the linearity ranges, slopes, intercepts, coefficients of determination, and limits of quantification/detection, for each analyte are reported in Table 2.

Calibration curves were fitted on linear regression models, with a 1/X weighing factor, to optimize the weight of the lowest concentration levels in the considered wide calibration ranges, with mean coefficients of determination higher than 0.997 for all the analytes.

In accordance with the guidelines, the LOD for each analyte was found considering a signal-to-noise (S/N) ratio greater than 3 times by dilution of the LLOQ (Table 2).

For all medications, the overall intra- and inter-day accuracies across all the QC levels were as follows: 103.0% and 109.3% for NVP, 104.8% and 98.2% for RPV, 108.2% and 109.2% for DTG, 98.5% and 107.7% for RAL, 105.5% and 103.0% for DRV, 108.2% and 104.9% for ATV, 105.9% and 104.7% for EFV, 105.9% and 114.1% for RTV, 105.0% and 109.1% for BIC, 101.4% and 103.1% for CAB, 98.7% and 99.7% for DOR, and 97.5% and 99.9% for TEM, respectively. The intra- and inter-day accuracy values calculated for each distinct QC’s concentration are reported in Table 3.

Moreover, the overall intra-day and inter-day precisions were evaluated and expressed in terms of RSD%: 5.0% and 10.8% for NVP, 5.2% 2.4% and 13.3% for RPV, 3.4% and 12.1% for DTG, 5.6% and 13.6% for RAL, 3.4% and 6.1% for DRV, 5.9% and 10.6% for ATV, 2.7% and 5.3% for EFV, 7.0% and 8.2% for RTV, 4.6% and 10.6% for BIC, 5.0% and 13.2% (1.4%) for CAB, 3.9% and 7.0% for DOR, and 6.8% and 12.9% for TEM, respectively. The details related to the intra- and inter-day imprecision of the method for each QC’s concentration levels are reported in Table 3.

Each criterion complied with FDA and EMA guidelines.

### 3.3. Recovery and Matrix Effect

Five replicates from six different plasma batches were spiked at three different concentration levels (QC-H, QC-M, and QC-L concentrations) to evaluate the drug recovery and matrix effect.

The absolute and the IS-n recovery and IS-n matrix effect values for each drug are reported in Table 4.

### 3.4. Stability

Stability in DPSs was assessed for each analyte by analyzing QC samples spotted on DSSDs and stored in foil bags with a desiccant, protected from light, after 1 week, 2 weeks, and 1 month at room temperature, and, additionally, after sequential storage for 1 week at room temperature and 1 week at 4 °C.

A graphical representation of the stability trends for each analyte is reported in the Supplementary Materials (Figure S1).

The results from the stability assessments are expressed as the percentage deviation from the freshly spotted QCs and are illustrated in Table 5.

### 3.5. Incurred Samples’ Re-Analysis and Method Comparison with Liquid Plasma Extraction

To assess the method performance in clinical practice, 38 real plasma samples from routine analysis were quantified and re-analyzed. In detail, the following was carried out: 8 patients were administered with BIC, 12 with RPV and CAB (LAI formulation), 12 patients with DRV, 8 with DTG, 5 with RTV, 4 with DOR, only 1 with EFV, and 1 with RAL.

The samples were analyzed and re-analyzed after one freeze-and-thaw cycle (known stability in liquid plasma) using DPSs; the mean RSDs % were 6.7% for BIC, 3.4% for RPV, 6.2% for CAB, 5.1% for DRV, 5.4% for RTV, 5.7% for DOR, 4.1% for EFV, 3.3% for DTG, and 1.4% for RAL. No sample showed an inter-day RSD higher than 20%, in accordance with EMA-ICH guidelines.

Moreover, the mean results from the DPS extraction were compared with the ones obtained from the currently in-use routine method for the quantification in liquid plasma. The low percent deviations confirmed the good accuracy and precision of the method. The detailed results are outlined in the Supplementary Materials (Tables S3 and S4).

For DTG, DRV, RPV, CAB, and BIC, Passing–Bablok regression and Bland–Altman plot analysis were performed for assessing the comparability of the two methods. The resulting plots for the above-listed medications are reported in the Supplementary Materials (Figure S2).

No significant over- nor under-estimation was observed for DTG, BIC, DRV, or CAB, while a slight but significant concentration-proportional overestimation was observed in DPSs for RPV.

## 4. Discussion and Conclusions

The extraction process ensures a clear extract and, despite the differences between the various medications, a reproducible recovery. The chromatographic run, lasting 15 min, allowed satisfactory chromatographic separation and reduced the probability of interference or the matrix effect. Co-elution was observed for RPV and TEM, despite their quite-different chemical features: despite this, no crosstalk nor mutual matrix effect were observed. Moreover, these drugs showed a comparable recovery and matrix effect, allowing us to use SIL-RPV as a good IS for TEM, as proven by the contained variability in the IS-normalized matrix effect and recovery. This is very advantageous, also from an economic point of view, considering the currently high cost of the isotope-labelled TEM.

The method was specific, since neither interfering peaks from matrix components, nor crosstalk between analytes or ISs were detected. Moreover, no carry-over was observed in “blank” samples analyzed after the injection of a high-concentration sample for any of the 12 drugs. LLOQ peak areas for each analyte were consistently 10 times higher than the mean background noise standard deviation of a blank injected after a high-concentration sample.

Accuracy and precision parameters resulted in compliance with FDA and EMA guidelines for method validation.

The calibration parameters (reported in Table 2) further confirmed the robustness of the calibration models.

Calibration ranges were chosen based on the expected drug concentrations in patients’ samples collected, at the steady state, at the end of the dosing interval (C_trough_, immediately prior to the next dose).

As reported in Table 4, the recovery and efficiency of extraction were low for some drugs (e.g., CAB) in terms of the mean percentages but, considering the low variability across the six different tested plasma lots and the use of matrix-matched calibration curves, this low recovery did not significantly impact the analytical results.

Both the mean matrix effect and IS-n matrix effect for each drug were contained and, most important, showed a very low variability (RSD always < 15%), demonstrating the non-significant influence of the matrix on the analytical results.

Similarly, the use of SIL-ISs effectively addressed the recovery and extraction efficiency issues for most analytes, markedly improving their percentages even in the most critical cases (e.g., RPV and RAL).

The stability results were optimal in DPSs for all molecules under the tested conditions (Table 5).

These data suggest that the analysis of DPSs with the proposed method is theoretically sensitive and reliable enough to be applied in the clinical context.

The analysis of real-world samples using DPSs demonstrated that the calibration ranges successfully cover the plasma concentrations for each drug, and their re-analysis confirmed the precision of measurements, with mean RSDs ranging from 1.4% to 6.7%.

Despite this, the lack of patients administered with NVP and ATV in our routine setting, explained by their infrequent use in Europe, prevents us from drawing a definitive conclusion about the incurred sample precision: nevertheless, this evaluation could be carried out in other regional contexts (e.g., developing countries), where NVP and ATV are still relatively widely used [44]. On the other hand, the lack of patients treated with TEM is explained by its too-recent introduction, with therapeutic indication limited to heavily treatment experienced patients.

Compared with previously published assays [20,21,22,23], the present method expands the panel of available drugs by including both less commonly used compounds (e.g., NVP and ATV) and newly introduced agents (e.g., TEM, RPV, and CAB in LAI formulations).

From a technical point of view, the described assay had results comparable with the previous ones in terms of linearity, and, owing to its good sensitivity and wide panel of tested drugs, it is well-suited to effectively address clinically relevant scenarios across different dosing regimens.

Additionally, compared with studies employing DBS, a better and more reliable correlation between DPS extraction and liquid plasma is expected. In fact, Weld et al. (2023) observed discordance between the results obtained with DBSs and liquid plasma for RPV and CAB, highlighting the need for a correction factor to reliably estimate the plasma concentration from DBS [23]. Nevertheless, the differential influence of hematocrit on the two drugs was also observed, possibly leading to different and variable correction factors for the different drugs. Summarizing, the use of DBS to estimate plasma concentrations involves a high dependency on the hematocrit, the characteristics of the considered molecules, and, theoretically, other genetic and non-genetic differences in drug-to-plasma ratio (e.g. depending on drug transporters, and protein binding, other than hematocrit) [45].

A method comparison with our in-use routine extraction from liquid human plasma was performed through Passing–Bablok regression and Bland–Altman analysis, which showed non-significant deviations from linearity (*p* > 0.05) for all the considered drugs and comparable results for DTG, CAB, BIC, and DRV, while RPV showed a significant tendency to overestimate the concentrations in DPS, compared to liquid plasma. This proportional overestimation (around 14%) is still within the acceptable accuracy thresholds and should not have an impact on the analytical performance, considering the very high expected intraindividual variability in the concentrations in the clinical real-life context (due to pharmacokinetics curves).

These findings appear to be sufficiently promising for the suitability of the method for TDM and pharmacokinetics (PK) research applications, especially regarding the newest LAI formulations, and useful for drug–drug interaction (DDI) management. The use of DPSs to collect samples could also be a valuable tool in TDM for monitoring patients’ adherence, still a quite-common issue between PLWH.

This work has some limitations: the application to real-life derived samples was limited to the most-used drugs and the Passing–Bablok and Bland–Altman evaluations showed slightly underpowered results for some drugs. Nevertheless, the validation parameters and the stability data advocate for the ruggedness of the method. The method has many advantages, such as easy and reliable spotting, the absence of a hematocrit effect, compared to DBS, a relatively simple and cheap extraction process, and reduced shipment and storage costs, as well as a reduced infectious risk associated with dried samples. Nevertheless, the DPS approach requires the separation of plasma before spotting (typically by centrifugation), and, in this work, we validated the analysis of plasma from venipuncture: a future study should evaluate the feasibility of the microsampling of capillary blood for ARV quantification and the possibility to couple this with DPS. On the other hand, from an environmental point of view, a drawback of this method is the use of hexane and dichloromethane during the extraction process, which are relevant environmental pollutants. Nevertheless, this drawback is counterbalanced by the feasibility of using very low volumes of plasma, allowing the use of low-volume sampling tubes (lower use of plastics and reduced costs), no need for disposable SPE cartridges, and the low carbon footprint deriving from the possibility of shipping samples at room temperature, avoiding the use of dry ice and reducing the overall weight of the shipment. Finally, it is worth noting that, compared to previous methods for the analysis of ARVs on DBS, which used Whatman 903 paper cards as support [21,22,23], we chose glass-fibre DSSDs. In fact, compared to paper, glass-fibre material is more chemically stable and it does not release the device’s components in the extraction solvents, thus reducing the risks of analytical interference, a matrix effect, or premature column clogging, or the need for MS source maintenance [46,47,48]. This is partially reflected, in this study, by the observed relatively contained and stable matrix effect.

Overall, this method is useful to empower PK studies and TDM in sites which are far from specialized laboratories, making it possible to considerably widen the availability of TDM to many hospitals and, potentially, foreign countries.

It is worth noting that other major obstacles are present in the wide-scale adoption of TDM for ARVs, mostly related to the limited agreement on therapeutic ranges, particularly for the most recently introduced drugs. In fact, to date, only the minimum effective concentration levels are defined for INSTI, based on the in vitro determined inhibitory concentrations, while there is no evidence of clearly toxic levels (also due to the good tolerability of these drugs) [49,50]. In this context, the use of DPSs could allow us to widen PK–PD studies in the real world to developing countries with high HIV prevalence, strongly increasing the statistical power to allow us to determine, confirm, and/or optimize therapeutic ranges [10,51].

Future studies are warranted to allow the evaluation of real-world performance.

## Figures and Tables

**Table 1:** General mass parameters (EV: Entrance Voltage, CC: Collision Cell voltage) and MRM ion traces for each molecule and SIL.

Analyte			Quantifier Trace			Qualifier Trace			Internal Standard (IS)
	Mode	RT (min)	*m*/*z*	EV	CC	*m*/*z*	EV	CC	
NVP	ESI+	2.42	267.1 > 226.1	18	−32	267.1 > 80.15	25	−56	SIL-RPV
TEM	ESI+	3.61	474.1 > 256.0	36	−30	474.1 > 215.1	27	−53	SIL-RPV
RPV	ESI+	3.65	367.3 > 195.2	45	−48	367.3 > 128.15	45	−57	SIL-RPV
SIL-RPV	ESI+	3.65	373.3 > 195.1	45	−46	373.3 > 129.15	45	−57	
CAB	ESI+	4.66	406.2 > 263.0	38	−32	406.2 > 363.1	38	−26	SIL-CAB
SIL-CAB	ESI+	4.66	410.2 > 366.1	39	−26	410.2 > 263.0	38	−32	
DTG	ESI+	5.54	420.2 > 277.1	26	−34	420.2 > 127.1	30	−46	SIL-DTG
SIL-DTG	ESI+	5.54	426.2 > 277.1	40	−35	426.2 > 133.1	40	−50	
RAL	ESI+	5.70	445.2 > 109.1	26	−41	445.2 > 361.1	26	−25	SIL-BIC
BIC	ESI+	6.04	450.2 > 289.05	42	−38	450.2 > 145.0	42	−44	SIL-BIC
SIL-BIC	ESI+	6.04	454.0 > 289.05	20	−38	454.0 > 148.0	18	−49	
DOR	ESI+	7.24	426.1 > 112.0	31	−35	426.1 > 315.0	31	−25	SIL-DOR
SIL-DOR	ESI+	7.24	432.1 > 112.0	31	−35	432.1 > 321.0	31	−25	
DRV	ESI+	8.21	548.3 > 392.2	17	−18	548.3 > 69.1	15	−66	SIL-DRV
SIL-DRV	ESI+	8.21	554.3 > 398.2	16	−19	554.3 > 69.1	15	−70	
ATV	ESI+	9.07	705.4 > 168.15	49	−64	705.4 > 144.15	40	−59	SIL-ATV
SIL-ATV	ESI+	9.07	711.45 > 168.1	40	−64	711.45 > 147.1	40	−59	
EFV	ESI−	11.33	314.0 > 68.9	−25	61	314.0 > 243.9	−25	22	SIL-EFV
SIL-EFV	ESI−	11.33	320 > 68.9	−24	61	320 > 249.9	−26	23	
RTV	ESI+	11.51	721.2 > 140.0	20	−87	721.2 > 296.0	20	−28	SIL-RTV
SIL-RTV	ESI+	11.51	725.2 > 140	20	−87	725.2 > 300	20	−28	

**Table 2:** Calibration parameters, limit of detection (LOD), and lower limit of quantification (LLOQ) for each analyte.

	Linearity Range (ng/mL)	Coefficient of Determination (R^2^)	Slope	Intercept	Limit of Detection (LOD)	Lower Limit of Quantification (LLOQ)
NVP	31.25–8000	0.998	0.05226	0.75718	10.42	31.25
RPV	9.77–2500	0.999	0.00884	0.02604	3.26	9.77
DTG	31.25–8000	0.999	0.00238	0.03379	10.42	31.25
RAL	11.72–3000	0.998	0.00866	0.02556	3.91	11.72
DRV	39.06–10,000	0.999	0.00199	0.04351	13.02	39.06
ATV	23.44–6000	0.999	0.00349	0.06596	7.81	23.44
EFV	62.50–16,000	0.999	0.00092	0.02993	20.83	62.50
RTV	9.77–2500	0.998	0.00820	0.08637	3.26	9.77
BIC	39.06–10,000	0.999	0.00289	0.06611	13.02	39.06
CAB	39.06–10,000	0.999	0.00303	0.04596	13.02	39.06
DOR	39.06–10,000	0.999	0.00281	0.05604	13.02	39.06
TEM	31.25–8000	0.998	0.04080	0.32916	10.42	31.25

**Table 3:** Precision and accuracy results from 5 replicates of each validation session at QC concentration levels (-H, -M, and -L), for each analyte.

	QC Level	Concentration (ng/mL)	Intra-Day Imprecision % (RSD)	Inter-Day Imprecision % (RSD)	Intra-Day Accuracy %	Inter-Day Accuracy %
NVP	H	6400	5.2	8.2	107.9	110.5
	M	1600	4.7	13.0	101.3	110.1
	L	160	5.1	11.3	99.8	107.4
RPV	H	2000	4.8	14.8	107.8	88.6
	M	500	5.8	11.9	103.1	93.8
	L	50	5.1	10.0	103.6	112.2
DTG	H	6400	3.9	12.2	114.4	110.4
	M	1600	3.0	13.3	108.8	110.8
	L	160	3.4	10.7	101.4	106.4
RAL	H	2400	6.2	11.5	98.0	112.5
	M	600	4.8	15.0	102.9	110.7
	L	60	5.9	14.2	94.5	99.8
DRV	H	8000	3.2	6.4	112.2	106.4
	M	2000	3.2	5.7	106.0	105.8
	L	200	3.9	6.3	98.2	96.9
ATV	H	4800	4.0	8.7	106.7	99.9
	M	1200	4.6	11.1	111.2	109.7
	L	120	9.1	11.9	106.7	105.1
EFV	H	12,800	2.3	5.9	101.9	103.5
	M	3200	2.8	5.8	110.1	109.5
	L	320	3.0	4.1	105.6	101.0
RTV	H	2000	6.4	7.2	109.7	106.5
	M	500	4.5	5.8	94.2	118.5
	L	50	10.0	11.5	113.9	117.4
BIC	H	8000	3.9	10.7	109.3	108.8
	M	2000	4.8	11.6	104.7	112.7
	L	200	5.1	9.5	101.0	105.7
CAB	H	8000	3.9	13.8	107.4	107.3
	M	2000	4.8	14.4	99.4	104.1
	L	200	6.4	11.3	97.3	97.8
DOR	H	8000	4.2	6.5	102.8	100.4
	M	2000	3.4	7.9	103.8	105.8
	L	200	4.2	6.5	89.4	92.8
TEM	H	6400	8.4	11.0	95.6	106.4
	M	1600	5.7	14.9	93.8	98.6
	L	160	6.4	12.9	103.1	94.7

**Table 4:** Mean recovery, matrix effect, extraction efficiency, and the IS-normalized (IS-n) validation parameters obtained from 5 replicates during 6 validation sessions, at each QC level (-H, -M, and -L), and for each analyte.

	QC Level	Recovery Mean % (RSD)	Matrix Effect Mean % (RSD)	Extraction Efficiency Mean % (RSD)	IS-n Recovery Mean % (RSD)	IS-n Matrix Effect Mean % (RSD)	IS-n Extraction Efficiency Mean % (RSD)
NVP	H	76.9 (4.1)	99.5 (1.3)	77.3 (4.4)	126.4 (11.1)	99.4 (0.9)	128.3 (10.8)
	M	72.7 (6.2)	98.6 (1.0)	73.8 (6.4)	120.6 (10.5)	99.3 (3.9)	121.5 (10.5)
	L	77.2 (8.9)	99.9 (1.5)	75.9 (4.0)	121.9 (10.7)	100.5 (4.0)	121.2 (9.5)
RPV	H	43.6 (14.9)	99.6 (2.5)	45.4 (14.8)	70.9 (14.6)	99.1 (2.8)	72.5 (14.4)
	M	44.9 (12.1)	99.4 (2.8)	44.4 (14.0)	71.0 (14.9)	98.0 (11.4)	71.0 (14.4)
	L	53.3 (4.8)	99.6 (6.0)	53.7 (8.3)	81.4 (10.5)	99.3 (5.9)	81.7 (9.5)
DTG	H	37.5 (12.1)	99.8 (1.5)	37.8 (12.5)	95.0 (9.0)	100.5 (1.5)	95.0 (9.1)
	M	29.4 (12.7)	101.1 (1.6)	29.8 (12.3)	93.9 (4.9)	100.4 (1.8)	94.4 (5.2)
	L	37.7 (9.5)	99.1 (2.4)	39.9 (14.3)	106.6 (4.7)	99.5 (2.9)	106.5 (5.0)
RAL	H	29.6 (14.2)	99.7 (1.1)	29.6 (14.9)	71.7 (12.2)	101.0 (2.0)	71.4 (12.1)
	M	25.1 (13.0)	100.4 (0.9)	25.1 (13.1)	76.0 (10.6)	101.1 (3.2)	74.7 (11.6)
	L	29.2 (14.3)	97.8 (2.5)	28.5 (13.0)	67.2 (7.8)	100.9 (4.8)	66.5 (9.2)
DRV	H	68.8 (10.8)	99.5 (1.7)	69.2 (12.0)	107.8 (4.2)	100.7 (1.7)	107.1 (4.5)
	M	64.4 (11.0)	100.9 (5.5)	63.8 (14.7)	108.8 (5.2)	100.5 (1.5)	108.8 (5.2)
	L	62.9 (14.6)	100.0 (3.3)	62.9 (13.9)	108.2 (7.7)	100.7 (3.5)	107.6 (8.0)
ATV	H	56.9 (10.5)	100.2 (1.5)	56.8 (10.4)	93.1 (7.0)	99.7 (2.2)	93.4 (6.9)
	M	51.2 (13.6)	101.0 (2.8)	50.2 (14.5)	109.1 (4.7)	99.7 (1.5)	108.2 (4.8)
	L	50.2 (14.9)	101.3 (1.0)	49.6 (13.2)	108.7 (9.9)	99.8 (1.1)	108.7 (9.6)
EFV	H	79.4 (13.2)	97.6 (0.9)	81.5 (13.8)	115.8 (13.9)	99.2 (5.2)	116.6 (12.7)
	M	71.0 (2.5)	98.8 (2.6)	72.0 (4.3)	102.3 (4.8)	100.0 (1.5)	102.3 (4.8)
	L	71.5 (8.6)	98.9 (1.0)	72.3 (8.1)	100.2 (5.9)	100.0 (2.0)	100.2 (6.2)
RTV	H	54.0 (12.5)	99.7 (1.8)	54.2 (13.2)	108.8 (7.3)	99.5 (4.2)	109.4 (7.7)
	M	45.2 (14.5)	99.4 (1.5)	45.4 (14.3)	123.1 (9.1)	101.0 (6.7)	121.3 (8.3)
	L	50.7 (9.7)	102.4 (2.3)	49.4 (10.2)	126.7 (12.0)	104.1 (8.5)	122.3 (13.7)
BIC	H	39.2 (13.1)	99.5 (1.3)	39.4 (13.9)	91.1 (4.9)	99.7 (1.9)	91.5 (4.7)
	M	29.8 (13.9)	99.5 (2.5)	30.1 (13.8)	93.6 (7.0)	99.8 (1.6)	93.2 (7.5)
	L	42.8 (11.3)	100.9 (0.8)	42.7 (11.7)	93.7 (6.1)	100.2 (4.5)	93.5 (5.9)
CAB	H	25.5 (9.8)	99.4 (2.2)	25.5 (9.7)	97.0 (6.8)	100.1 (2.5)	97.1 (6.7)
	M	22.7 (13.7)	95.9 (11.3)	22.9 (14.8)	92.8 (6.6)	101.4 (2.2)	93.7 (4.8)
	L	25.0 (13.6)	102.0 (2.7)	24.3 (14.6)	93.0 (8.8)	99.9 (4.0)	93.2 (7.0)
DOR	H	57.5 (9.7)	99.0 (1.2)	58.1 (10.1)	101.7 (7.1)	9.4 (2.0)	102.3 (6.9)
	M	50.6 (10.2)	100.0 (3.4)	50.7 (12.6)	104.8 (8.5)	99.8 (1.8)	105.0 (8.2)
	L	48.3 (14.9)	98.6 (1.5)	49.0 (14.2)	98.8 (4.9)	100.6 (2.8)	99.4 (7.0)
TEM	H	65.5 (11.1)	99.5 (1.7)	65.9 (12.5)	111.7 (10.7)	98.9 (4.0)	113.3 (12.1)
	M	55.5 (14.9)	98.4 (1.7)	56.4 (14.9)	102.2 (14.2)	98.7 (4.7)	104.5 (14.2)
	L	58.1 (13.9)	100.5 (5.3)	57.6 (13.9)	96.4 (13.1)	99.7 (4.0)	96.8 (12.5)

**Table 5:** Stability results expressed as % deviation from the freshly spotted sample for each analyte at each QC level after 1 and 2 weeks at room temperature (RT), and 2 weeks (one at RT plus 1 at 4 °C), and 1 month at RT.

		Deviation from the Freshly Spotted Sample, %											
		NVP	RPV	DTG	RAL	DRV	ATV	EFV	RTV	BIC	DOR	CAB	TEM
QC H	1 week RT	−7	2	−19	7	3	3	−1	−18	−7	−11	−11	10
	2 weeks RT	4	9	−2	1	−5	22	4	8	5	−8	−2	1
	2 weeks (RT + 4 °C)	−4	−1	−2	7	−6	−8	−6	8	−2	0	−3	−9
	1 month RT	2	−11	0	10	−10	−17	−12	−15	−6	−4	−1	7
QC M	1 week RT	−1	13	−18	15	5	3	−2	−4	−5	−24	−22	4
	2 weeks RT	−1	22	1	7	3	22	17	10	11	−12	−10	−6
	2 weeks (RT + 4 °C)	−7	−8	3	2	−3	−1	−1	−6	3	−3	−14	4
	1 month RT	2	−24	8	9	−12	−5	13	5	−3	15	19	22
QC L	1 week RT	10	16	−8	11	6	5	5	−12	−12	−33	−29	2
	2 weeks RT	−15	16	−1	7	3	18	7	−4	0	−20	−9	−8
	2 weeks (RT + 4 °C)	−1	−34	7	−3	−18	−13	6	13	−12	−21	−19	16
	1 month RT	14	−4	14	16	−10	−2	12	14	26	25	31	23

## Data Availability

The data presented in this study is contained within the article and Supplementary Material. Further inquiries can be directed to the corresponding author.

## Supplementary Materials

The following supporting information can be downloaded at: https://www.mdpi.com/article/10.3390/pharmaceutics18040513/s1, Table S1: Chromatographic gradient for elution over time. F.A. = Formic Acid; Table S2: General detector settings; Figure S1: Stability trends for DPS-DSSDs at each QC level after 1 and 2 weeks of RT storage, 2 weeks (one at RT plus one at +4 °C) and 1 month at RT storage; Table S3: Real-world samples mean concentrations (ng/mL) for each drug following DPS-DSSD extraction and RSDs %; Table S4: Mean percentage deviations between the in-use routine method for plasma concentration and DPS-DSSD method; Figure S2: Passing–Bablok regressions and Bland–Altman plots for DTG, DRV, RPV, CAB, and BIC.

## Abbreviations

The following abbreviations are used in this manuscript:

FDA	Food and Drug Administration
EMA	European Medicines Agency
DDI	Drug–Drug Interaction
DSSD	Dried Sample Spot Device
DPS	Dried Plasma Spot
DBS	Dried Blood Spot
TDM	Therapeutic Drug Monitoring
UHPLC-MS/MS	Ultra-High Performance Liquid Chromatography–Tandem Mass Spectrometry
NVP	Nevirapine
DTG	Dolutegravir
DRV	Darunavir
RPV	Rilpivirine
RAL	Raltegravir
EFV	Efavirenz
RTV	Ritonavir
ATV	Atazanavir
BIC	Bictegravir
DOR	Doravirine
CAB	Cabotegravir
TEM	Temsavir
RT	Retention Time
IS	Internal Standard
SIL	Stable Isotope Linked
LLOQ	Lower Limit of Quantification
LOD	Limit of Detection
QC	Quality Control
-H	High
-M	Medium
-L	Low
STD9	Standard 9
ESI+	Positive electrospray ionization
RSD	Relative Standard Deviation
ACN	Acetonitrile
MeOH	Methanol
H_2_O	Water
F.A.	Formic Acid
DMSO	Dimethyl Sulfoxide
IS-nME	IS-normalized matrix effect
IS-nREC	IS-normalized recovery
MRM	Multiple Reaction Monitoring
S/N	Signal-to-Noise
