# Supplementary material for: UHPLC–MS/MS Method for the Simultaneous Quantification of 12 Antiretroviral Drugs in Human Plasma Using Dried Sample Spot Devices: Development, Validation, and Stability Evaluation
Source: Pharmaceutics. 2026 Apr 21;18(4):513. doi: 10.3390/pharmaceutics18040513 (PMC13119037; doi:10.3390/pharmaceutics18040513)
Supplement: Supplementary file 1 [file pharmaceutics-18-00513-s001.zip › pharmaceutics-4208470-supplementary.pdf]

Supplementary Materials

Table S1. Chromatographic gradient for elution over time. F.A. = Formic Acid.

Time (min)	Flow (mL/min)	**Phase A %** (H_2O + F.A. 0.05 %)	**Phase B %** (ACN + F.A. 0.05 %)
0.00	0.4	70	30
0.30	0.4	70	30
8.00	0.4	53	47
9.00	0.4	45	55
10.00	0.4	40	60
11.00	0.4	25	75
11.70	0.4	5	95
13.20	0.4	5	95
13.25	0.4	70	30
15.00	0.4	70	30

Table S2. General detector settings.

**General detector settings**	
**Electrospray V Pos/Neg (V)**	5300/3500
**Nebulizer gas flow (L/h)**	350
**Source 1 Temperature (°C)**	350
**Heating gas flow (L/h)**	350
**Multiple 1 RF**	430
**Drying gas (L/h)**	130
**HSID Temperature (°C)**	280
**Multiple 1 RF**	430
**Collision Pressure (AU)**	410

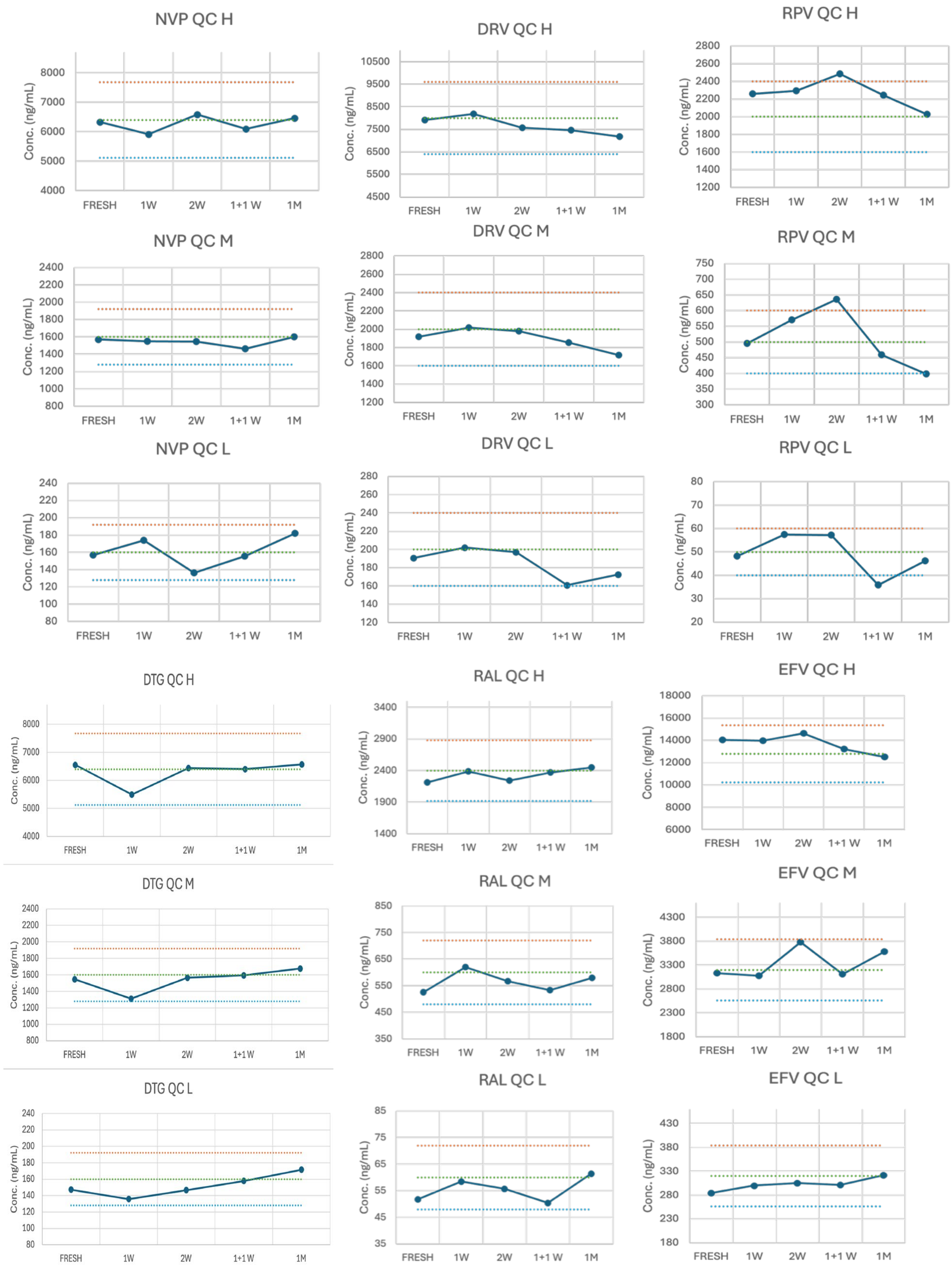
NVP QC H
DRV QC H
RPV QC H
NVP QC M
DRV QC M
RPV QC M
NVP QC L
DRV QC L
RPV QC L
DTG QC H
RAL QC H
EFV QC H
DTG QC M
RAL QC M
EFV QC M
DTG QC L
RAL QC L
EFV QC L
Conc. (ng/mL)
FRESH
1W
2W
1+1 W
1M

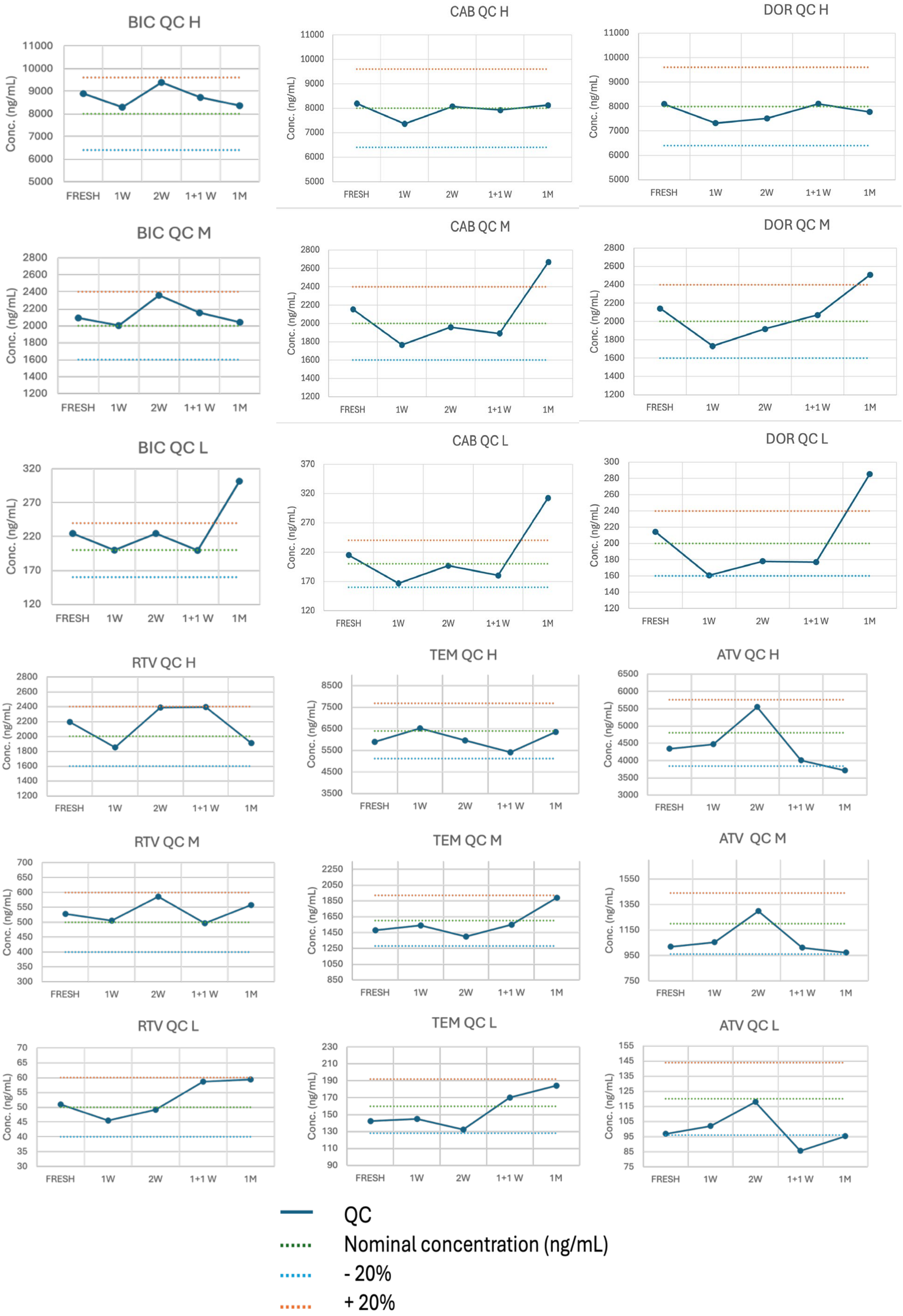

Figure S1. Stability trends for DPS-DSSDs at each QC level after 1 and 2 weeks of RT storage, 2 weeks (one at RT plus one at +4°C), and 1 month at RT storage

Table S3. Real-world samples mean concentrations (ng/mL) for each drug following DPS-DSSD extraction and RSDs %.

	DPS-DSSD mean conc. [ng/mL] (RSD%)								
	**BIC**	**RPV**	**CAB**	**DRV**	**RTV**	**DOR**	**EFV**	**DTG**	**RAL**
Sample 1	3596 (2.3)								
Sample 2		130 (2.1)	3424 (17.3)						
Sample 3	4558 (3.3)			2340 (4.5)	154 (5.4)				
Sample 4		78 (8.2)	3197 (4.2)						
Sample 5				3182 (0.1)	156 (5.0)	1801 (8.3)			
Sample 6		94 (3.1)	447 (3.3)						
Sample 7	5346 (7.0)								
Sample 8		123 (0.0)	3176 (2.7)						
Sample 9						605 (4.9)			
Sample 10				2355 (1.7)					
Sample 11						615 (4.5)			
Sample 12							1121 (4.1)		
Sample 13				4914 (6.5)	315 (6.3)				
Sample 14				2451 (10.5)				1686 (8.6)	
Sample 15								772 (1.9)	
Sample 16								2256 (2.9)	
Sample 17									48 (1.4)
Sample 18	2645 (19.6)								
Sample 19		100 (0.6)	1287 (0.9)						
Sample 20				3588 (7.2)					
Sample 21		69 (9.3)	783 (1.8)						
Sample 22		71 (3.0)	2558 (5.5)						
Sample 23	3501 (8.8)								
Sample 24				4140 (4.9)	127 (7.8)				
Sample 25				807 (8.5)				101 (1.4)	
Sample 26		53 (4.0)	1073 (9.2)						
Sample 27	1866 (4.2)								
Sample 28				919 (8.8)				311 (2.0)	
Sample 29	1537 (4.6)								
Sample 30		105 (0.7)	1643 (3.3)						
Sample 31				4753 (2.5)	1260 (2.7)			2980 (2.3)	
Sample 32		149 (1.9)	1806 (14.2)						
Sample 33						3338 (5.0)		5770 (1.3)	
Sample 34		110 (6.4)	1819 (7.0)						
Sample 35		191 (3.7)	3033 (5.3)						
Sample 36				2226 (2.0)					
Sample 37	1039 (3.9)								
Sample 38				1596 (4.3)				1296 (6.0)	

Table S4. Mean percentage deviations between the in-use routine method for plasma concentration and DPS-DSSD method.

	In-use routine vs DPS-DSSDs extraction method mean dev. %								
	**BIC**	**RPV**	**CAB**	**DRV**	**RTV**	**DOR**	**EFV**	**DTG**	**RAL**
Sample 1	6%								
Sample 2		2%	31%						
Sample 3	-11%			2%	-10%				
Sample 4		-3%	-2%						
Sample 5				-5%	2%	5%			
Sample 6		3%	-8%						
Sample 7	6%								
Sample 8		2%	-8%						
Sample 9						-7%			
Sample 10				7%					
Sample 11						-10%			
Sample 12							2%		
Sample 13				-10%	-11%				
Sample 14				-6%				3%	
Sample 15								3%	
Sample 16								-6%	
Sample 17									-14%
Sample 18	-28%								
Sample 19		8%	-6%						
Sample 20				0%					
Sample 21		-6%	-19%						
Sample 22		-7%	-23%						
Sample 23	-5%								
Sample 24				-1%	1%				
Sample 25				-2%				-5%	
Sample 26		-14%	-5%						
Sample 27	-3%								
Sample 28				-3%				4%	
Sample 29	3%								
Sample 30		-4%	1%						
Sample 31				-8%	-1%			-6%	
Sample 32		17%	-1%						
Sample 33						9%		4%	
Sample 34		1%	-3%						
Sample 35		1%	-16%						
Sample 36				1%					
Sample 37	-2%								
Sample 38				0%				-2%	

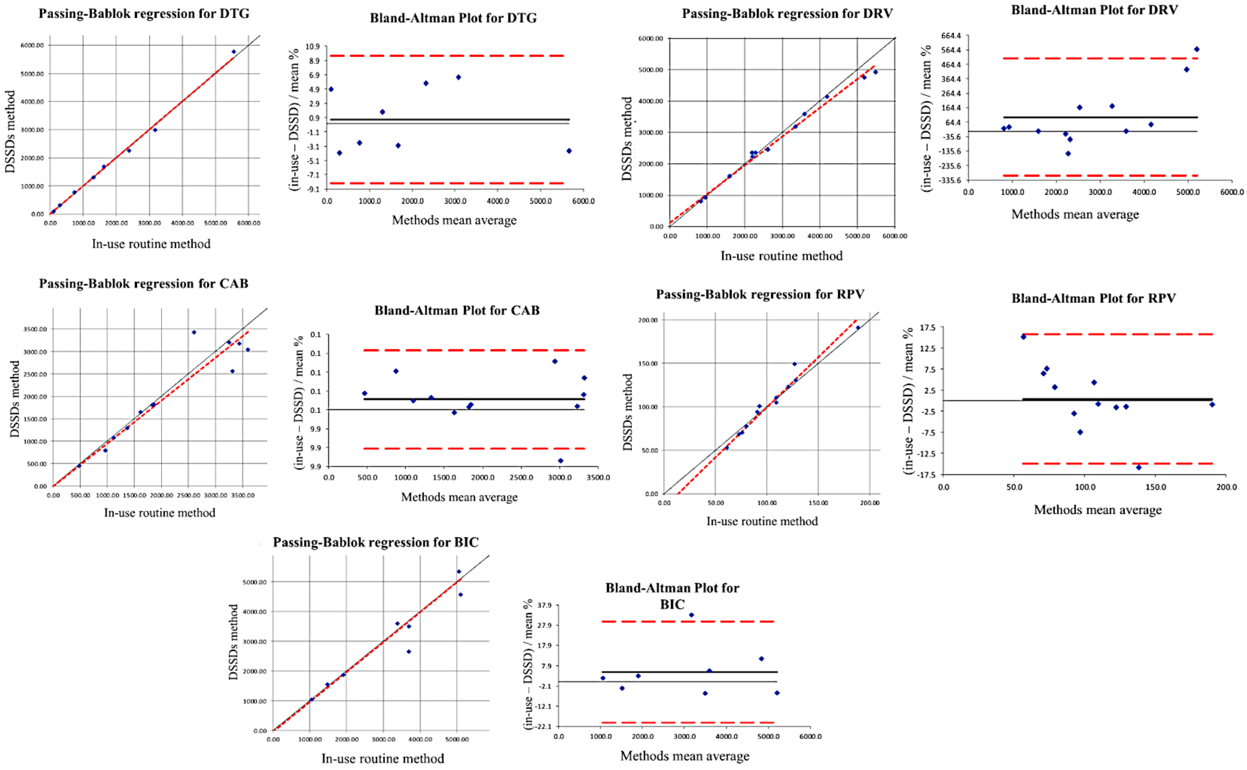

Figure S2. Passing-Bablok regressions and Bland-Altman Plots for DTG, DRV, RPV, CABO, and BIC.
Passing-Bablok regression 95% CIs: -19.1 – 88.2 for DTG; -27.2 – 374.2 for DRV; -273.4 – 363.3 for CABO; -40.0 - -3.0 for RPV; -1269.8 – 564.1 for BIC.
Bland-Altman 95% CIs: -8.3 – 9.5 for DTG; -7.3 – 11.9 for DRV; -20.4 – 31.8 for CABO; -15.0 – 15.7 for RPV; -20.1 – 29.9 for BIC.
